# COVID-19 Detection from Computed Tomography Images Using Slice Processing Techniques and a Modified Xception Classifier

**DOI:** 10.1155/2024/9962839

**Published:** 2024-05-24

**Authors:** Kenan Morani, Esra Kaya Ayana, Dimitrios Kollias, Devrim Unay

**Affiliations:** ^1^ Izmir Democracy University Uckuyular, Gursel Aksel Blv No: 14 35140, Karabaglar, Izmir, Türkiye; ^2^ Yildiz Technical University Yildiz 34349 Besiktas, Istanbul, Türkiye; ^3^ Queen Mary University of London 327 Mile End Rd, London, UK

## Abstract

This paper extends our previous method for COVID-19 diagnosis, proposing an enhanced solution for detecting COVID-19 from computed tomography (CT) images using a lean transfer learning-based model. To decrease model misclassifications, two key steps of image processing were employed. Firstly, the uppermost and lowermost slices were removed, preserving sixty percent of each patient's slices. Secondly, all slices underwent manual cropping to emphasize the lung areas. Subsequently, resized CT scans (224 × 224) were input into an Xception transfer learning model with a modified output. Both Xception's architecture and pretrained weights were leveraged in the method. A big and rigorously annotated database of CT images was used to verify the method. The number of patients/subjects in the dataset is more than 5000, and the number and shape of the slices in each CT scan varies greatly. Verification was made both on the validation partition and on the test partition of unseen images. Results on the COV19-CT database showcased not only improvement from our previous solution and the baseline but also comparable performance to the highest-achieving methods on the same dataset. Further validation studies could explore the scalability and adaptability of the developed methodologies across diverse healthcare settings and patient populations. Additionally, investigating the integration of advanced image processing techniques, such as automated region of interest detection and segmentation algorithms, could enhance the efficiency and accuracy of COVID-19 diagnosis.

## 1. Introduction

The unprecedented global challenge posed by the COVID-19 pandemic has underscored the critical need for advanced diagnostic methodologies to effectively curb the virus's spread. Among these methodologies, computed tomography (CT) imaging has emerged as a vital tool in providing detailed insights into the manifestations of the disease. In this context, the utilization of CT scan images has proven instrumental in detecting the presence of the virus and understanding its impact on the respiratory system. The intricate details captured by CT scans offer a comprehensive view of the pulmonary structures, making them invaluable for early and accurate diagnosis [1].

To address the urgency of timely and precise COVID-19 diagnosis, the integration of advanced computational techniques has become imperative. Deep learning, particularly through the lens of transfer learning, has demonstrated remarkable potential in enhancing diagnostic accuracy and efficiency. Transfer learning, a paradigm that leverages pretrained models to expedite the learning process, plays a pivotal role in the analysis of medical images. In the realm of COVID-19 diagnosis, these approaches contribute significantly by automating feature extraction and pattern recognition, thereby streamlining the diagnostic workflow [2].

The importance of employing deep learning, and specifically transfer learning, lies in its ability to decipher complex patterns within CT images associated with COVID-19 manifestations. By building on knowledge gained from related tasks, transfer learning models quickly adapt to the unique characteristics of COVID-19 pathology. This not only expedites the diagnostic process but also enhances the accuracy of identifying subtle nuances in CT scan images indicative of viral infection. The potential of these approaches to revolutionize COVID-19 diagnosis underscores the need for continued research and development in this domain [3].

Building upon our previous methodologies [4], the new proposed solution seeks to further elevate the accuracy of COVID-19 diagnosis through a refined approach. We choose to keep the solution light and avoid more deep learning incorporation. Therefore, we focus on processing the data before inputting it into a modified and lean Xception model. This innovative preprocessing strategy is aimed at addressing specific challenges encountered in CT scans, such as nonrepresentative slices, by systematically removing them. Moreover, the proposed method involves manual cropping of images to retain only the pertinent lung areas in each slice, focusing the analysis on regions critical for COVID-19 detection. The rationale behind this image processing technique is to enhance the effectiveness of the subsequent transfer learning model. By providing the model with refined and relevant input data, we aim to optimize its performance in discerning COVID-19-related patterns.

In this paper, we present a literature review in Section 2. We then move on to Section 3 to present the methodology focusing more on our proposed image processing techniques and quickly discussing our modified Xception referring to our previous paper on that. Section 3 also mentions the COV19-CT database used for our study. In Section 4, we introduce the results of our new solution, show the improvement that we achieved in comparison to our previous study, and compare it to the baseline performance and other alternatives. In Section 5, we make our conclusion and discuss the study's limitations building the possibility of future research directions.

## 2. Literature Review

In recent years, the application of CT scan image processing techniques has gained substantial attention in the realm of COVID-19 diagnosis. These techniques prove useful in keeping the solution lightweight by replacing deep learning models with data preprocessing. Researchers have explored innovative approaches to enhance the accuracy of detection models by strategically manipulating CT scan slices. A noteworthy study in [5] came second on the leader of the workshop aimed at COVID-19 diagnosis. The authors discussed the significance of selectively removing uppermost and lowermost slices from chest CT scans. They came to leave only 40% of the slices at the center of each patient's CT scan image. The rationale behind this technique was to focus on central slices, optimizing the dataset for COVID-19 detection. Their findings demonstrated that this slice removal method contributed to a more precise localization of relevant anatomical regions, laying the groundwork for improved diagnostic performance.

Further, investigations into the manual cropping of CT scan slices have yielded promising outcomes. In an earlier study of ours [6], manual cropping was employed to accentuate lung areas in chest CT images. The study recognized the importance of emphasizing regions crucial for COVID-19 detection, and manual cropping emerged as a viable solution. By systematically resizing each slice to specifically highlight the lung areas, the study reported heightened sensitivity and accuracy in identifying COVID-19-related patterns. These approaches showcased the impact of focusing on the region of interest in the images and image segmentation.

To measure the efficiency of using slice processing techniques on the final diagnosis of COVID-19, a challenging database, named “COV19-CT-DB,” was utilized by many researchers [7–12].

The baseline approach in [7] proposed a CNN-RNN model, comprising a CNN part for local 2D slice analysis and an RNN part for sequential analysis of the entire 3D scan. The model outputs probabilities for each CT scan slice, and the final diagnosis is determined by a voting scheme, specifically an “at least one” approach. If at least one slice predicts COVID-19, the entire CT scan is diagnosed as COVID-19; if all slices predict non-COVID-19, the whole CT scan is diagnosed as non-COVID-19. Furthermore, preprocessing of the images involves extracting CT images, clipping voxel intensity values, and normalizing. The methodology utilizes ResNet50 transfer learning as the CNN model, followed by a GRU layer in the RNN model. The macro F1 score achieved was 0.96 on the test partition. ResNet transfer learning network is considered heavy compared to other transfer learning models, given the number of parameters that it has to train.

Another study that utilized a transfer learning approach selected 3D ResNet architecture to propose a solution on the same database [13]. A CT scan classifier employing three-dimensional convolutional neural networks (CNNs) is based on the ResNet 3D 18 model. The preprocessing step standardizes the number of slices in CT scans, reducing resolution sizes through bicubic interpolation. The model was fine-tuned for COVID-19 detection and severity classification, and adjustments include input channel modifications and dropout integration. The macro F1 score achieved on the test partition was 0.878. The study could also be considered heavy using the ResNet model in 3D-shaped image input.

The second highest macro F1 score on the test set came from the work that introduced the CMC-COV19D network for COVID-19 diagnosis. It combines contrastive representation learning (CRL) and mix-up classification. In the CRL process, CT scans undergo stochastic data augmentation, encoding, projection to a low-dimensional vector, and classification. The contrastive loss function distinguishes positive and negative pairs among augmented samples. Mix-up classification enhances model generalization, creating mix-up samples and labels during training [14].

The highest-achieving method [15] came with a two-step approach. Initially, they use conventional backbone networks for extracting semantic feature embeddings from each CT scan slice. Subsequently, they introduce a long short-term memory (LSTM) and transformer-based subnetwork to facilitate temporal feature learning, resulting in spatiotemporal feature representation. This two-step LSTM model effectively prevents overfitting and enhances performance. The two-step LSTM model excels in minimizing false negatives, while the 2-step Swin model excels in minimizing false positives. The use of heavy Swin transformers and other deep learning methods leads to a nonlean solution in this study, albeit a definite great performance.

Finally, our previous Xception-based solution [4] came with a macro F1 score of 0.82, proposing a lean transfer learning-based solution. We kept the solution easy to follow by minimally processing the input images. The Xception model performed better when compared to other heavier transfer learning alternatives, namely, VGG16, introducing a lighter transfer learning-based solution.

In recent years, the utilization of CT scan image processing techniques has garnered significant attention in the domain of COVID-19 diagnosis. These techniques offer a means of maintaining a lightweight solution by focusing on data preprocessing rather than employing complex deep learning models. Notably, our previous research explored the efficacy of manual cropping to highlight lung areas in chest CT images, demonstrating heightened sensitivity and accuracy in identifying COVID-19-related patterns. Furthermore, the strategic removal of nonrepresentative slices from CT scans has shown promise in optimizing datasets for COVID-19 detection. To assess the impact of these slice processing techniques on the final diagnosis of COVID-19, researchers have turned to challenging databases such as the “COV19-CT-DB.” Previous studies have employed various methodologies, including transfer learning approaches utilizing heavyweight models like ResNet50, which, while effective, may pose computational burdens due to their large parameter sizes. In contrast, our proposed solution leverages the Xception model, chosen for its lightweight architecture and comparative accuracy. By incorporating slice processing techniques into our methodology, we aim to enhance diagnostic performance while maintaining computational efficiency. This approach represents a strategic balance between simplicity and effectiveness, offering a promising avenue for advancing COVID-19 diagnosis using CT images.

## 3. Methodology

The method follows in two parts. Our previous solution did not include the image processing part in the solution. This method is aimed at adding this part before using a classifier for diagnosing the disease (https://github.com/IDU-CVLab/COV19D_2nd).

### 3.1. Image Processing

In the pursuit of refining the input data for our classifier, we implemented two key image processing techniques, each designed to bolster the model's accuracy and minimize misclassifications at the patient level. It is worth noting about the COV19-CT database used in our study that medial preprocessing details of the slices such as normalization were not provided to the competing teams when sharing the data. Rather, the images in the database were received mainly in loosely compression format: Joint Photographic Experts Group (JPEG) format, grayscale images, with 8-bit depth.

Firstly, selective slice removal was applied. Our first image processing technique involves the judicious removal of slices from each CT scan, strategically aimed at preserving only those slices that distinctly represent COVID-19 manifestations. Specifically, we systematically eliminate 40% of the slices in each CT scan, removing an equal number of uppermost and lowermost slices. This curation ensures that the retained slices are central and, therefore, more likely to encapsulate the characteristic features of COVID-19 pathology within the patient. By discarding nonrepresentative slices, we intend to enhance the model's focus on the most relevant sections of the CT scan, thereby contributing to a more accurate and nuanced classification. This selective slice removal process aligns with the overarching goal of tailoring the input data to the unique characteristics of COVID-19 presentations in each patient. Recognizing that the upper and lower extremes of CT scans may not consistently capture the crucial features indicative of the virus, our approach optimizes the dataset to foster a more precise and targeted analysis.

**Figure 1:**
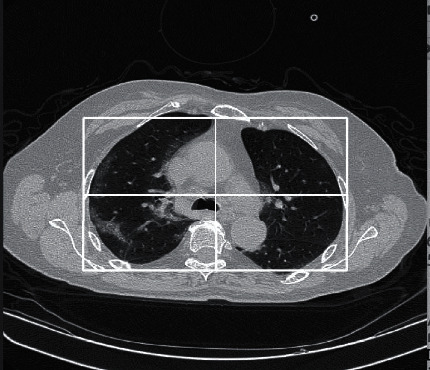
Rectangular cropping of the slices.

Secondly, Manual Cropping for Lung Area Emphasis was applied. The second facet of our image processing strategy involves manual cropping of all slices, transitioning from the original 512 × 512 dimensions to a standardized size of 227 × 300.  Figure 1 shows the resulting cropped slices from the original images. This deliberate resizing is not merely an arbitrary adjustment; rather, it is a meticulous act aimed at emphasizing the lung areas within each slice. The manual cropping was made so that the lung areas in the representative slice, i.e., not the uppermost and lowermost slices of the CT scan, are both in the resulting images. These slices are the slices that will be left after the slice removal techniques and the cropped areas are the areas of interest showing the virus infection.  Figure 1 shows the cropping made on a representative slice. The resulting images are of the size 227 × 300. By focusing on the anatomical regions most pertinent to COVID-19 detection, we facilitate the classifier in honing its attention to the key structures indicative of the viral infection. The choice to manually crop each slice aligns with the understanding that the nuances of COVID-19 pathology often manifest prominently in the lung areas. This deliberate act of slice cropping enhances the classifier's ability to discern subtle patterns associated with the virus, ultimately contributing to heightened diagnostic accuracy.

Finally, all slices were resized to 224 × 224, adding more channels to reach 3 as a standard input image for the Xception model. The reason behind resizing again is that the Xception model expects input of the same dimensions. In addition, the workstation capabilities for sufficient low training time as well as common practicality indicate 224 × 224 to be the selected input image dimensions.

### 3.2. Modified Xception Model Classifier

**Figure 2:**
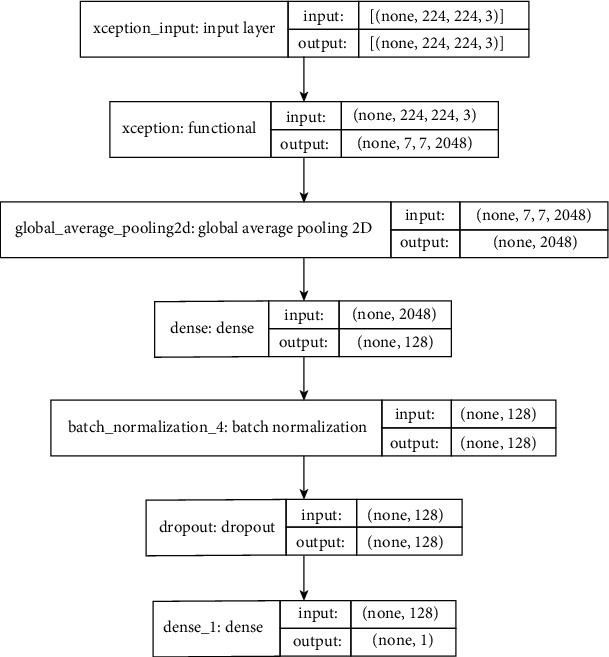
Xception model architecture (adapted for our task).

In our previous methodology in [4], we used an Xception model [16] with a modified output to make final diagnostic decisions.  Figure 2 shows the Xception model architecture with the adapted output.

The Xception model architecture employed in our methodology comprises a series of layers designed to extract features from input data. Initially, a global average pooling layer aggregates feature maps across spatial dimensions, reducing computational complexity while preserving relevant information. Subsequently, a dense layer with 128 filters and rectified linear unit (ReLU) activation facilitates nonlinear transformations, enhancing the model's capacity to capture complex patterns in the data. Batch normalization is applied to stabilize and accelerate the training process by normalizing activations within each minibatch. A dropout layer with a rate of 0.2 is incorporated to prevent overfitting by randomly deactivating a fraction of neurons during training. Finally, a dense layer with a sigmoid activation function outputs the class probability of a slice being a non-COVID-19 case. The final layer's output represents the class probability of being a non-COVID-19 case slice. This class probability is then compared against a predefined threshold, determining the slice's classification as COVID-19 or non-COVID-19. These individual slice-level determinations collectively lead to patient-level diagnoses, as explained in the latter sections of the paper. Several class probability thresholds were explored to optimize performance, and their effects were evaluated on the validation set of the COV19-CT database.

**Figure 3:**
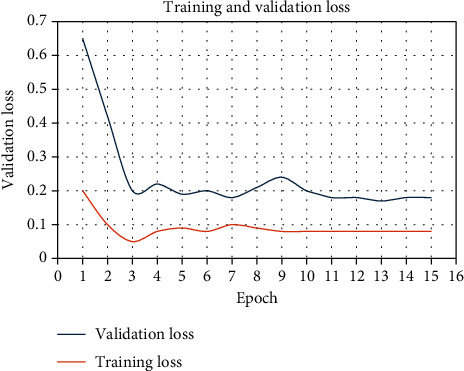
Training and validation loss.

For model compilation, we employed the Keras platform [17], utilizing the “Adam” optimizer initialized with a learning rate of 0.001. The loss function was set as “binary cross entropy.” Our model was trained across 15 epochs, a determination that emerged from rigorous experimentation. During these trials, it was observed that further increasing the epoch count resulted in only marginal improvements in validation loss over a prolonged timeframe.  Figure 3 shows training and validation loss during the training period.

Training the CNN model, with a batch size of 32, across the epochs necessitated approximately 10 days of computation. This was facilitated on a workstation operating a GNU/Linux system, equipped with 64 GiB of system memory and powered by an Intel(R) Xeon(R) W-2223 CPU @ 3.60 GHz processor. These specifications offer insights into the computational resources and time investment involved in achieving our model's refined performance for COVID-19 detection.

In our training process, we employed transfer learning models with a 3-channeled input, utilizing pretrained weights from the ImageNet model. Notably, we rendered the model's weights nontrainable during our training phase to preserve the pretrained features. To enhance the training dynamics, we implemented callback mechanisms, specifically utilizing the “ReduceLROnPlateau” callback. This mechanism is a dynamic learning rate adjustment strategy commonly used in deep learning model training. It operates by continuously monitoring a specified metric, in our case, the validation loss. If the validation loss does not improve over a predefined number of training epochs (in this case, 2 epochs), the learning rate is adjusted downward by a certain factor. This approach was particularly useful in optimizing the performance of our COVID-19 detection model, considering the complex and multifaceted nature of COVID-19-related patterns in CT images. By adaptively adjusting the learning rate when stagnation occurred in the validation loss, we aimed to facilitate model progress through challenging regions of the loss landscape.

### 3.3. The Dataset

The dataset utilized in this investigation is an extension of the COV19-CT-DB, playing a crucial role by providing a comprehensive collection of CT scans essential for the detection of COVID-19. This dataset encompasses a significant number of CT scans, encompassing 1,650 instances of COVID-19 and 6,100 non-COVID-19 cases. This balanced distribution facilitates a robust assessment of the performance of the proposed method across different classes.

What distinguishes the “COV19-CT-DB” dataset is not only its size but also its diversity, encompassing variations in the number of cases and the variability in COVID-19 manifestations. The CT scans in the dataset have been meticulously labeled by a panel of experts, each possessing over 20 years of experience, ensuring the accuracy and reliability of the labels, crucial for the construction and evaluation of machine learning models. The consensus of the annotation came with 98% agreement. Furthermore, polymerase chain reaction (PCR) test results were used for confirmation in the annotation of the images.

The dataset's diversity, spanning a spectrum of COVID-19 and non-COVID-19 cases, introduces unique challenges and opportunities. Given that COVID-19 exhibits a range of manifestations, capturing this variability becomes vital for the development of an effective detection model. The inclusion of cases with varying degrees of lung involvement and diverse clinical presentations in the “COV19-CT-DB” dataset mirrors the real-world complexity of COVID-19 instances.

With its comprehensive labeling, extensive size, and diversity, the “COV19-CT-DB” dataset serves as an ideal foundation for assessing the effectiveness of the proposed method. Its suitability arises from its capacity to rigorously evaluate the model's performance on diverse cases, ensuring not only accuracy but also robustness in identifying COVID-19 instances within varying clinical contexts. Each CT scan consists of a variable number of slices, ranging from 50 to 700, and access to this dataset is facilitated through the “ECCV 2022: 2nd COV19D Competition.” Table 1 illustrates the distribution of COVID-19 and non-COVID-19 cases for our study.

### 3.4. Performance Evaluation

The proposed model was evaluated via the COV19-CT-DB database using accuracy, macro F1 score, and confidence interval. Accuracy, recall, precision, and macro F1 score are fundamental metrics used to evaluate the performance of classification models, including our COVID-19 detection model. Accuracy represents the proportion of correctly classified instances out of the total instances, providing an overall measure of the model's correctness. Recall, also known as sensitivity, measures the model's ability to correctly identify positive instances out of all actual positive instances. Precision, on the other hand, quantifies the proportion of correctly identified positive instances out of all instances classified as positive by the model, reflecting the model's ability to avoid false positives. The macro F1 score combines precision and recall into a single metric, considering both false positives and false negatives, thereby providing a balanced assessment of the model's performance across different classes [18]. On the other hand, confidence intervals, as calculated for the reported validation accuracy scores, offer insights into the reliability and variability of the results. They indicate the range within which the true accuracy of the model is likely to fall, providing a measure of uncertainty around the reported performance metric. A narrower confidence interval suggests higher confidence in the reported accuracy score, while a wider interval indicates greater uncertainty. Understanding the implications of confidence intervals is crucial for interpreting the robustness and generalizability of the model's performance beyond the specific dataset used for validation. Thus, by providing context and interpretation for these performance metrics and confidence intervals, we aim to enhance the readers' understanding of the model's evaluation and contribute to a more informed assessment of its reliability and variability [19]. Below are the equations for the performance measuring matrices that are added:

The accuracy is calculated as in

(1) $$\begin{array}{c} \text{Accuracy}=\frac{\text{True positives}+\text{true negatives}}{\text{True positives}+\text{false positives}+\text{true negatives}+\text{false negatives}}, \end{array}$$

where positive and negative cases refer to COVID-19 and non-COVID-19 cases.

The macro F1 score was calculated after averaging precision and recall matrices as in

(2) $$\begin{array}{c} \text{Macro }F1=\frac{2\times \text{average precision}\times \text{average recall}}{\text{average precision}+\text{average recall}}, \end{array}$$

where the precision and recall for classification tasks are defined as in

(3) $$\begin{array}{c} \text{Precision}=\frac{\text{True positive}}{\text{True positive}+\text{false positive}}, \end{array}$$

(4) $$\begin{array}{c} \text{Recall}=\frac{\text{True positive}}{\text{True positive}+\text{false negative}}. \end{array}$$

The confidence intervals were used to check the range variance of the reported results. The residuals of the interval can be calculated as in

(5) $$\begin{array}{c} \text{Radius of interval}=z\times \sqrt{\frac{\text{value considered}\times \left(1-\text{value considered}\right)}{n}}, \end{array}$$

where *z* is the number of standard deviations from the Gaussian distribution and *n* is the number of samples.

## 4. Results

The results of our methodology are discussed on the validation set both at the slice level and at the patient level.

### 4.1. Results at Slice Level

Table 2 shows the training performance for different metrics.

To calculate the confidence interval for the resulting validation accuracy score (0.8848), equation (3) was used. In the equation, *z* is taken as *z* = 1.96 for a significance level of 95%. By that, we can obtain the confidence interval keeping in mind that the number of samples (slices) in the validation set is 30235, to be approximately 0.0036. With that, the validation accuracy score can be said to be 0.8848 ± 0.0036.

**Figure 4:**
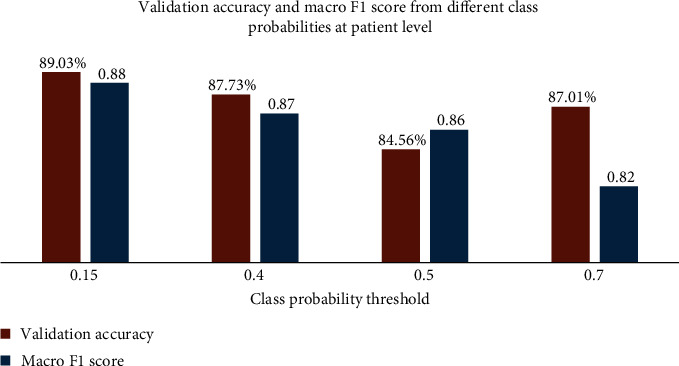
Model performance against different class probability thresholds on the validation set.

Using the above-mentioned method, predictions were made through different class probability thresholds. These thresholds are compared to the model output. The model has only one output, which is the probability of the slice being a non-COVID-19 slice. After that, the majority voting method for each CT scan was deployed to decide whether the patient belonging to that CT scan was COVID-19 positive or not.  Figure 4 shows performance results on the validation set at the patient level for four different class probability thresholds. The comparison was made in terms of the validation accuracy and the macro F1 score.

The findings indicate that, among the three suggested class probability thresholds, the 0.15 threshold level gives the best performance. This holds when considering both validation accuracy and validation macro F1 score. Consequently, our proposed approach exceeds the baseline model score, as reported in [5], in terms of macro F1 score, achieving a score of 0.88 on the validation set.

**Figure 5:**
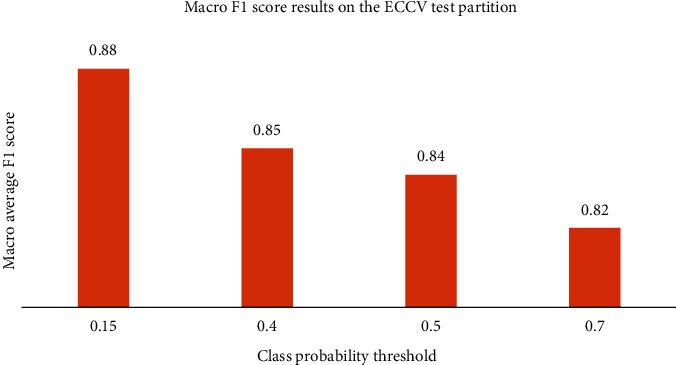
Macro average F1 score results on the test partition for different class probability threshold.

To further validate our results, we test the method on the test partition of unseen images. The partition is named “ECCV Partition” from the COV19-CT database. The highest macro F1 score achieved is 0.88. Similar to our results on the validation set, the highest macro F1 score is achieved with a class probability threshold of 0.15. With that, the threshold to be selected for further comparison and evaluation is at 0.15 threshold.  Figure 5 shows the results of the test partition.

Our previous method, which was proposed without image preprocessing, reached only 0.82 macro F1 score on the ECCV test partition. These results indicate that our extended method far exceeds the previous one.

Further, our macro F1 score is now comparable to more alternative solutions that are on the leaderboard as can be seen in Table 3. The table shows our proposed method compared to the highest-achieving solutions, and the solutions used a transfer learning approach on the same test partition.

In an attempt to understand the model's misclassification at the patient level, we conducted a statistical analysis [20] given the number of misclassified slices and the number of all slices for each patient in the validation set, the COVID-19-labeled cases.  Table 4 shows the average percentage of misclassification happening in the targeted dataset. The table also shows the correlation between the two values. The statistical study was conducted at a 0.7-class probability threshold.

## 5. Discussion

From the statistical results of misclassification of COVID cases, we can conclude that for every COVID-19 misclassified patient, some numbers of slices were correctly classified. We also find a highly positive correlation between the number of slices in each CT scan and the number of misclassifications. In other words, the increased number of slices increases misclassification and vice versa. All in all, tuning the voting method/threshold at the patient level can help optimize the method's results to the needs of the clinics of hospitals. For example, voting on considering the patient to be a COVID-19 patient if 20% of their slices were predicted as COVID-19 would mean that more patients will be considered COVID-19 positive than otherwise they would with a majority voting approach.

## 6. Conclusion and Further Work

In conclusion, we have extended our previous method by adding image processing techniques to CT scan slices before classification. The image processing techniques included uppermost and lowermost slice removal in each CT scan and manual rectangular cropping to the original slices to focus on the lung areas. For classification, our method uses the same transfer learning approach that we introduced in our previous study, a modified Xception model classifier. Proposing the image processing techniques in this paper gave better performance compared to our previous solution, the baseline solution, and many other alternatives on the same dataset. With that, we propose an accurate and lean solution.

The limitation of this study lies in the manual choice of the percentage at which central slices are kept in each CT scan. In this study, we considered leaving 60% percent of representative slices for each patient. However, using this percentage can give better performance results. On this note, the authors wish to further their studies by tuning this percentage to produce better performance for the solution.

Another limitation lies in the manual cropping of the images to keep the lung area. This cropping may give better performance if tunned, or if proper segmentation of the lung areas was deployed. This shall be another gap for improvement and research to be conducted in the future.

## Figures and Tables

**Table 1:** Distribution of cases in the COV19-CT database.

Annotation	Training data	Validation data
COVID-19 CT cases	882	224
Non-COVID-19 CT cases	1110	468

**Table 2:** Performance results of the training at the slice level.

Performance metric	Score
Average training accuracy	97.30%
Average validation accuracy	88.48%
Average recall	0.917
Average precision	0.909
Macro F1 score	0.891

**Table 3:** Average macro F1 score results from the comparison of validation and test partitions.

The method	Test set
ACVLab [15]	0.891
FDVTS [14]	0.891
MDAP [13]	0.878
Our previous method (IDU-CVLab) [4]	0.82
Baseline	0.690
Our extended method (with a 0.15 threshold)	0.880

**Table 4:** Statistical analysis of the number of misclassifications for each patient.

Number of all COVID-19 cases in the validation set	224
Average percentage of misclassification	16.47%
Max percentage of misclassification	60.81%
Min percentage of misclassification	13.89%
Correlation between the number of misclassified slices and all number of slices	0.998

## Data Availability

Data are provided and should be ordered from the corresponding entity as stated in the “Methodology” section and “Dataset” subsection.

## Additional Points

The code related to this study can be found on GitHub at https://github.com/IDU-CVLab/COV19D_2nd.
